# Dr. William V. Blighton

**Published:** 1918-02

**Authors:** 


**Dr. William V. Blighton** of North Tonawanda died Dec. 16, after a lingering illness. He was born at Varysburg, Sept. 16, 1837. He entered Columbia College and later graduated from Greene Co. Homeopathic College. He practiced medicine until 1874 when he was called to the ministry of the Methodist Episcopal church. He returned to the practice of medicine in 1874 when he moved to North Tonawanda and practiced there until his death.

